# Onkopus: precise interpretation and prioritization of sequence variants for biomedical research and precision medicine

**DOI:** 10.1093/nar/gkaf376

**Published:** 2025-05-16

**Authors:** Nadine S Kurz, Kevin Kornrumpf, Tim Tucholski, Klara Drofenik, Alexander König, Tim Beißbarth, Jürgen Dönitz

**Affiliations:** Department of Medical Bioinformatics, University Medical Center Göttingen, 37077 Göttingen, Germany; Göttingen Comprehensive Cancer Center (G-CCC), 37075 Göttingen, Germany; Department of Medical Bioinformatics, University Medical Center Göttingen, 37077 Göttingen, Germany; Department of Medical Bioinformatics, University Medical Center Göttingen, 37077 Göttingen, Germany; Institute of Pathology, University Medical Center Göttingen , 37075 Göttingen, Germany; Department of Medical Bioinformatics, University Medical Center Göttingen, 37077 Göttingen, Germany; Göttingen Comprehensive Cancer Center (G-CCC), 37075 Göttingen, Germany; Department of Gastroenterology, Gastrointestinal Oncology and Endocrinology, University Medical Center Göttingen, 37075 Göttingen, Germany; Department of Medical Bioinformatics, University Medical Center Göttingen, 37077 Göttingen, Germany; Göttingen Comprehensive Cancer Center (G-CCC), 37075 Göttingen, Germany; Campus Institute Data Science (CIDAS), Section Medical Data Science (MeDaS), 37077 Göttingen, Germany; Department of Medical Bioinformatics, University Medical Center Göttingen, 37077 Göttingen, Germany; Göttingen Comprehensive Cancer Center (G-CCC), 37075 Göttingen, Germany; Campus Institute Data Science (CIDAS), Section Medical Data Science (MeDaS), 37077 Göttingen, Germany

## Abstract

One of the major challenges in precision oncology is the identification of pathogenic, actionable variants and the selection of personalized treatments. We present Onkopus, a variant interpretation framework based on a modular architecture, for interpreting and prioritizing genetic alterations in cancer patients. A multitude of tools and databases are integrated into Onkopus to provide a comprehensive overview about the consequences of a variant, each with its own semantic, including pathogenicity predictions, allele frequency, biochemical and protein features, and therapeutic options. We present the characteristics of variants and personalized therapies in a clear and concise form, supported by interactive plots. To support the interpretation of variants of unknown significance (VUS), we present a protein analysis based on protein structures, which allows variants to be analyzed within the context of the entire protein, thereby serving as a starting point for understanding the underlying causes of variant pathogenicity. Onkopus has the potential to significantly enhance variant interpretation and the selection of actionable variants for identifying new targets, drug screens, drug testing using organoids, or personalized treatments in molecular tumor boards. We provide a free public instance of Onkopus at https://mtb.bioinf.med.uni-goettingen.de/onkopus.

## Graphical Abstract

**Graphical Abstract:**
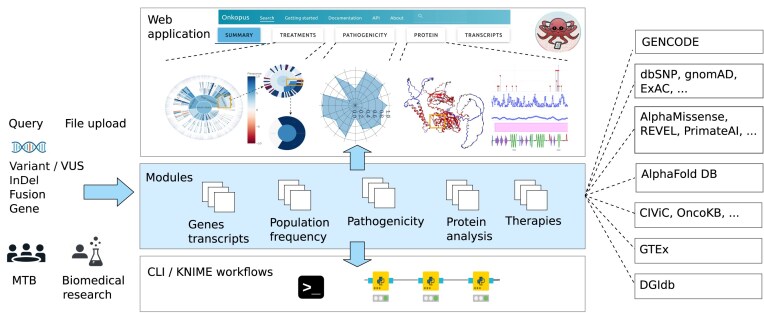

## Introduction

The rapid advancements of next-generation sequencing have led to an enormous expansion of genomic data, presenting challenges in identifying disease-causing variants and translating these findings into effective targeted therapeutic interventions. Accurate interpretation of variant pathogenicity and clinical significance is essential for precision medicine and biological research, as it enables the development of targeted therapies and enhances our understanding of disease mechanisms and pathways. However, an obstacle to the interpretation of variants in clinical practice is the need to manually query numerous databases and aggregate the results. Variant annotation pipelines aim to close this gap by automating the process of annotating variant data. Existing annotation pipelines, including ANNOVAR, SnpEff, and the Variant Effect Predictor (VEP) [1–3], primarily focus on the functional annotation of genetic variants. Several web-based tools, such as REEV and VarCards2 [4, 5], primarily support the biological interpretation of variants but do not focus on matching therapy suggestions or evaluating all variants at the level of a cell line or patient. On the other hand, there are platforms that have specialized in clinical interpretation, such as PanDrugs2 and MTB-Report [6, 7], suggesting potential treatments for pathogenic variants. For clinical use with cancer patients, some platforms provide solutions for variant interpretation in preparation for a molecular tumor board (MTB), such as MTB Portal, cBioPortal or PredictONCO [8–10], or commercial solutions, such as VarSome [11] or Illumina Connected Insights (ICI).

Another aspect that has been little considered in the context of variant interpretation yet is the analysis of the protein-specific context of a variant and the molecular characteristics of the amino acid exchange that may contribute to variant pathogenicity. There are tools that are able to analyze the impact of mutations on the protein structure [12] or on binding affinity [13] but do not provide a comprehensive variant interpretation. Each tool has its own focus and strengths, typically emphasizing either biological or clinical interpretation, with few approaches covering all aspects.

Here we present a powerful yet intuitive framework designed to support the biological and clinical interpretation of genetic variants and prioritize the most pathogenic ones. Our approach includes several steps to support initial variant selection and analysis, ranging from high-confidence evidence derived from established studies to predicted effects on protein structure. Our framework places a special emphasis on annotating and analyzing the molecular and structural consequences of protein-coding variants. To address the different user requirements, we provide our tool as a software package, an interactive web application, and customizable workflows for the analytical framework KNIME [14].

## Materials and methods

### Modules and API endpoints

Onkopus consists of three main building blocks: the Onkopus modules, the server, and the web front end. The modules provide the core functionality for annotating variants. Additional functions, such as the parsing of queries and uploaded files, querying the modules and aggregating the results, and generating interactive graphics, are provided by the Onkopus server. All modules were implemented using downloaded databases, ensuring Onkopus, except the OncoKB module, is not dependent on third-party web services or software. The modules’ web services were built using Python (version 3.11) and the Flask package. Table-formatted databases, including dbNSFP, REVEL, and CIViC, were indexed using tabix [15]. For database files based on CSV and GTF format (GENCODE, DGIdb), SQL-based database tables were generated from the source files using pymysql and SQLAlchemy. For the Genotype-Tissue Expression (GTEx) gene expression dataset, we used SQLite 3 to generate an indexed database.

The web front end (Onkopus Web) was implemented using Vue.js v3, AG Grid, and a SwaggerUI API (application programming interface) documentation. Interactive radar plots, sunburst graphs, and protein feature visualizations were implemented using Plotly [16]. Chromosome locations were plotted with Ideogram.js. 3D protein structures were visualized using 3Dmol.js [17]. Genome sequencing data were visualized by integrating the Integrative Genomics Viewer (IGV) genome browser [18]. Requests are accelerated by caching requests using a Redis server. All Onkopus modules are containerized using Docker [19]. A complete list of all data sources integrated in Onkopus is provided in Supplementary Table S1.

### Genomic, transcript, and protein data

To convert genomic, transcript, and protein notations, we integrated SeqCAT [20] as a module. Functional regions and transcripts were retrieved from GENCODE v47 [21]. Variant data parsing, reading and writing files, variant validation in HGVS (Human Genome Variation Society) and VCF (Variant Call Format) notation, variant type recognition, and liftover transformations between genome assemblies were implemented using AdaGenes. Onkopus works internally on hg38 (GRCh38); other reference genomes, including hg19 (GRCh37) and T2T-CHM13, are utilized using LiftOver. Median gene-level transcripts per million normalized gene expression data were obtained from the GTEx project [22].

### Clinical evidence

For retrieving evidence-based data on possible personalized therapies, CIViC [23], MetaKB [24], and OncoKB [25] were integrated into Onkopus. OncoKB offers different licenses depending on whether the use is intended for an academic or commercial purpose. If the results of the optional OncoKB module are to be integrated, a valid OncoKB license is thus required. To aggregate data from all three databases, we extracted a set of features from each database, including the associated biomarker, cancer type, drugs, response type, evidence level, evidence statement, and citation ID. To merge duplicates among clinical evidence databases, treatments were merged if the normalized features were identical (drug label, cancer entity, evidence level, response type, and citation ID). Drug classifications were retrieved from the DrugOn ontology, a drug classification system based on manual and automated classifications. To calculate the evidence level of a variant’s clinical significance, we used the mapping of database-specific evidence codes to the AMP/ASCO/CAP (Association for Molecular Pathology, American Society of Clinical Oncology, and College of American Pathologists) guidelines [26] proposed by Wagner *et al.* [24]. To harmonize the proposed treatments, CIViC treatments with multiple drugs whose therapy interactions were labeled as “substitutes” were converted into separate treatment options. To normalize the data regarding the sensitivity of targets, we generated a custom mapping for each database. For CIViC, all values of the “significance” column were extracted that contain “Sensitivity” or “Resistance.” For OncoKB, the response types “R1” and “R2” were mapped as resistant, and “1,” “2,” “3A,” and “3B” as sensitive. As for MetaKB, the descriptive value of the evidence association was mapped as response type. To normalize cancer types, we extracted the main tumor types defined by OncoTree [27]. We then applied a combination of exact and fuzzy matching to map cancer types to the OncoTree categories.

### Variant effect predictions

Data for modules on variant effect predictions and population allele frequency data were extracted from dbNSFP v4.9c [28] and dbSNP [29], including variant pathogenicity predictions of AlphaMissense [30], REVEL [31], PrimateAI [32], ESM1b [33], SIFT [34], and others. The ClinVar database [35] was downloaded for clinical reports.

### Molecular and protein features

To compute different features of a mutated site’s protein, we downloaded AlphaFold-predicted protein structures of wild-type MANE Select transcripts [36, 37]. To provide binding site predictions, we computed binding sites of the AlphaFold-predicted protein structures by using ScanNet [38]. The relative accessible surface area (RSA) and the secondary protein structure were computed with DSSP (Dictionary of Protein Secondary Structure) [39]. The RSA of a protein residue quantifies the proportion of the amino acid’s surface that is exposed to the surrounding environment accessible surface area (ASA) compared to a fully extended reference state maximum possible solvent accessible surface area (MaxASA), calculated as


(1) \begin{eqnarray*}
{\rm RSA} = \frac{{\rm ASA}}{{\rm MaxASA}}.
\end{eqnarray*}

The *C*_α_ distances were calculated using the Euclidean distance between each amino acid’s $C_{\alpha _{i}}$ atom and the $C_{\alpha _{m}}$ atom position of the mutated amino acid:


(2) \begin{eqnarray*}
d_{C_{\alpha _{i}}} = \sqrt{(x_i - x_m)^2 + (y_i - y_m)^2 + (z_i - z_m)^2}.
\end{eqnarray*}

Protein domain information included in the Pfam database [40] has been retrieved using InterproScan 5 [41]. BLOcks SUbstitution Matrices (BLOSUM62) scores representing alignments between evolutionary divergent protein sequences were computed using the Python blosum package. Molecular features of amino acids and protein sequences were calculated using Biopython.

### Variant classification

Onkopus prioritizes variants according to the standardized guidelines of the American College of Medical Genetics and Genomics (ACMG) guidelines [42], which classify variants into five categories based on their pathogenicity (pathogenic, likely pathogenic, benign, likely benign, and unknown significance). Onkopus performs the classification by evaluating the retrieved annotations of a variant based on the ACMG rules.

## Results

### Functionality of the Onkopus web server

We present Onkopus, a comprehensive framework for the biological and clinical interpretation of genetic variants. Onkopus utilizes the advantages of a modular architecture: Each module provides API endpoints, which follow a common pattern for syntax and semantic.

**Figure 1.**
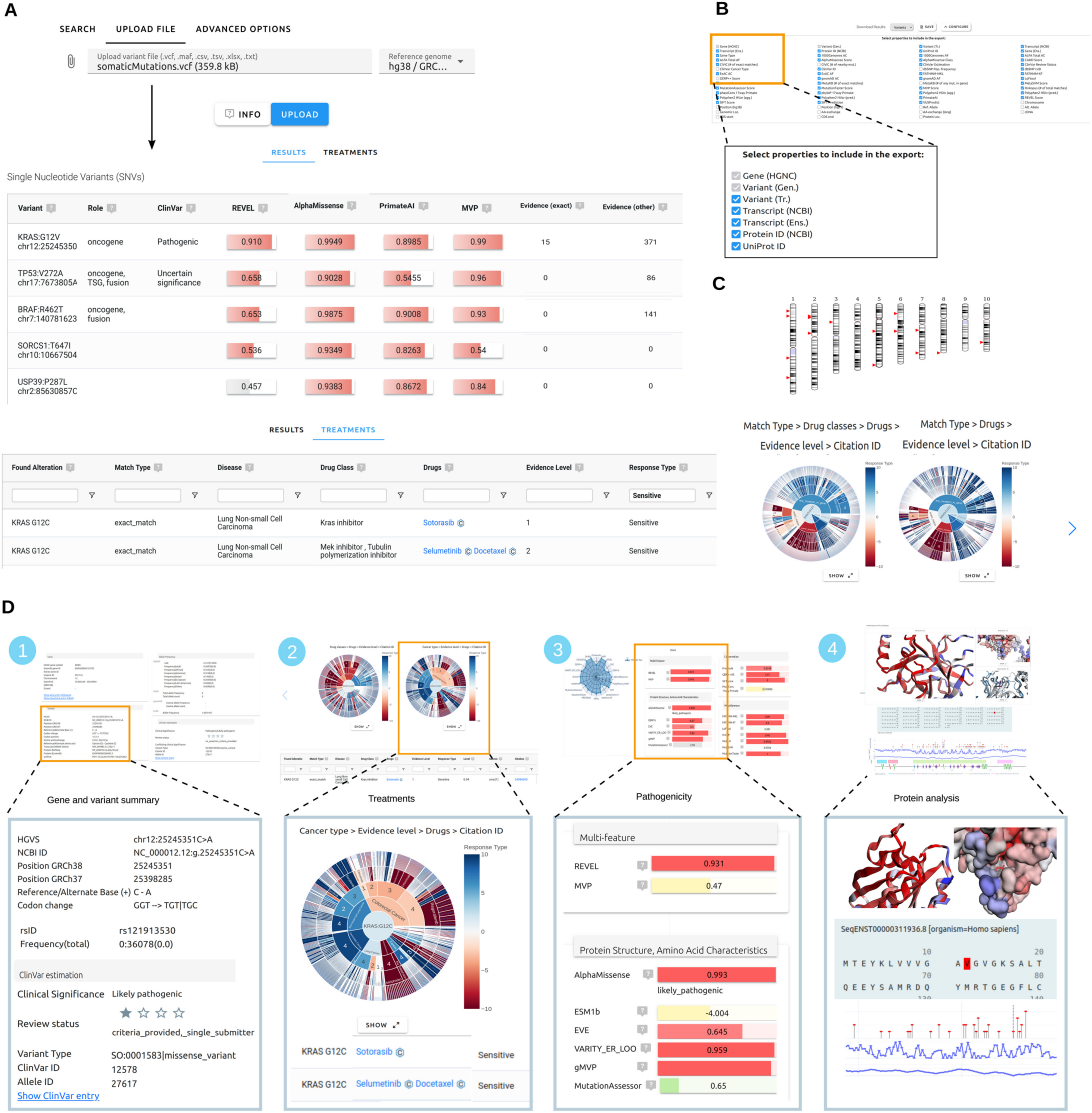
The Onkopus web front end. (**A**) The input can be a variant search or an uploaded variant file at the DNA, transcript, or protein level. After the processing, Onkopus presents a result table listing the annotated variants for single nucleotide variants, insertions and deletions, gene fusions, and genes ranked according to the ACMG classification. A second table presents potential treatments and the biomarker’s clinical significance, listing the associated biomarker, the match type, drugs, drug classification, evidence level, the response type, and the citation ID of the underlying publication for each study. (**B**) The annotated variant data and potential treatments are both downloadable as CSV files, with the option to choose which features are to be exported. (**C**) Visualizations showing the chromosomal locations of variants and sunburst plots of potential personalized therapies. (**D**) Detailed pages of a single-nucleotide variant, including (1) genetic details, (2) clinical significance and potential treatments, (3) pathogenicity predictions, and (4) protein analysis, among others.

Variant data can be provided in various formats from DNA to protein level. A special feature of Onkopus is its ability to parse queries in different variant nomenclatures at DNA (e.g. “chr12:g.25245350C>T”), transcript (“NM_004985.5:c.35G>A”), and protein level (“KRAS:p.G12D”) in common file formats like VCF or MAF (Mutation Annotation Format). After submitting a query or file upload, Onkopus runs the variant interpretation and presents two result tables: the variant table listing the annotated variants, and a treatments table presenting potential personalized therapies for the molecular profile (Fig. 1A). In addition, the option to download variants and therapies (Fig. 1B), as well as to explore the results in interactive visualizations (Fig. 1C), is available. In addition, each variant may be analyzed individually by clicking on the variant’s “Details” button: For single nucleotide variants (SNVs), we provide a comprehensive overview of details on genomic and transcriptomic characteristics, clinical evidence, variant effect predictions, molecular and protein analysis, transcripts, and gene expression (Fig. 1D). The variant table sorts variants by default according to the highest estimated pathogenicity, based on the ACMG guidelines classification. The variants may also be sorted according to other features, including ClinVar reports, pathogenicity predictions by REVEL, AlphaMissense or PrimateAI, or according to clinical significance with regard to potential therapies. The tabs on the detail page reflect the steps of interpreting variants as it is practiced in MTBs: (i) obtaining general information about the gene and variant, (ii) analyzing evidence-based treatments, (iii) estimating the variant pathogenicity, and (iv) inspecting the mutation’s impact on the protein structure and characteristics, transcripts, and gene expression. Similarly, we provide detailed pages for insertions and deletions, gene fusions, and the entire gene. All links are persistent, allowing the results of a variant interpretation to be bookmarked or shared.

### Variant- and gene-level implications

The first tab of the variant details page provides background information about the selected variant and the corresponding gene and protein. We provide general information about the affected gene, including function, oncogene or tumor suppressor classification, the genomic location, transcripts, functional regions, population allele frequency, ClinVar reports, and CIViC summaries. In addition, we provide links to additional resources or information, including GeneCards [43], CIViC, dbSNP, ClinVar, Ensembl, and VarSome.

In addition to single nucleotide variants and copy number variations, Onkopus supports the analysis of gene fusions by specifying the chromosomes and breakpoints. The tool is capable of providing information regarding the position of the gene and the determination of whether the fusion is in-frame. Onkopus classifies a variant’s estimated pathogenicity according to the ACMG guidelines. Users can see how the classification was calculated on the “Classification” tab of each variant’s details page. Genes can also be analyzed as a whole, including genetic data, transcripts, known variants, and therapies. We offer a more expansive annotation scope than previous solutions and provide new features, including calculations and annotations on molecular and structural protein features (Supplementary Table S2).

In the following, we will demonstrate how to use Onkopus for two use cases: First, we will show how our framework helps in finding a personalized therapy for a molecular profile. Then, we will show how variants of unknown significance (VUS) can be interpreted by analyzing the protein-specific context of variants.

### Targeted therapies

**Figure 2.**
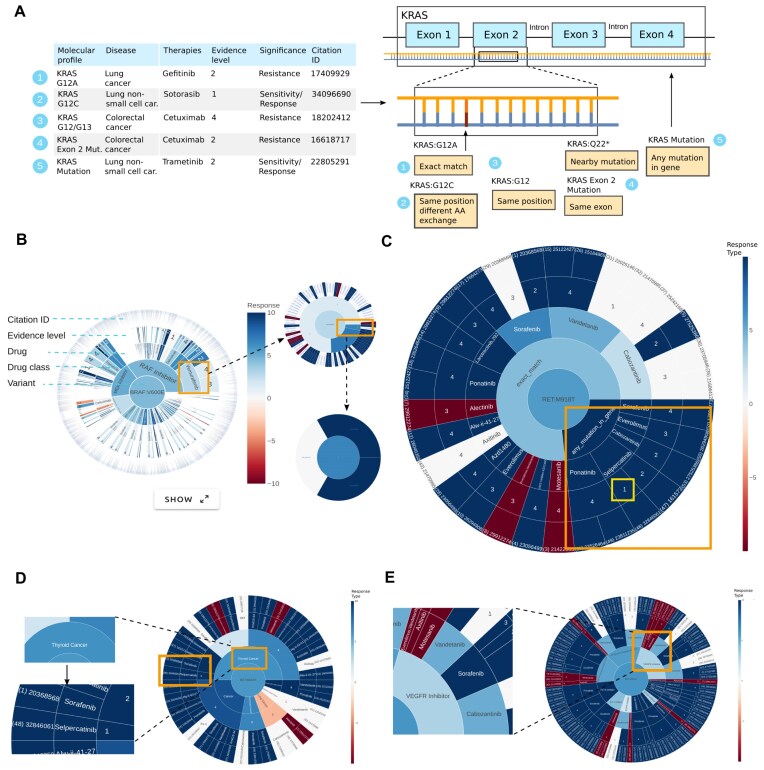
Therapy overview and sunburst visualization of personalized therapies in Onkopus. (**A**) Match types in Onkopus for the search for potential treatments for a specific biomarker, including (1) exact matches (e.g. “KRAS G12A”), (2) evidence for variants with differing amino acid exchange at the same position (“KRAS G12C”), (3) variants at the same position with an arbitrary exchange (“KRAS G12”), (4) variants within the same exon, or (5) clinical evidence on targeting the whole gene (“KRAS mutation”). (**B**) Interactive sunburst visualization of the clinical significance of a biomarker regarding potential treatments. The sunburst visualization allows even large numbers of treatment options to be explored by clicking on a cell and triggering a zoom into the selected area. The outermost layer of each graph comprises the linked citation IDs, representing the number of evidences. The color coding represents the response type of the retrieved clinical evidence, with red indicating resistance, blue indicating sensitivity, and white indicating unknown. (**C**) Sunburst visualization of potential treatments for RET:p.M918T visualized according to the sequence “match type” > drug > evidence level > citation ID, from inner to outer layer. The graph is colored according to response type. In this case, we can find a sensitive therapy, selpercatinib (yellow box), based on the “any mutation in gene” (orange box) match type with evidence level 1. (**D**) Personalized therapies for RET:p.M918T visualized according to cancer type within the first layer, and (**E**) according to drug classification.

Onkopus is able to find a large number of possible therapies for a variant by searching the integrated databases for various suitable associated biomarkers, so-called match types. In the first step, Onkopus searches for exact matches where the associated biomarker of the clinical evidence aligns precisely with the variant of the patient (e.g. “BRAF V600E”) (Fig. 2A). In the next step, it searches for matches with differing base substitutions at the same genomic position (“BRAF V600R”), as well as any mutation in the gene (“BRAF mutation”). The same evidences from different sources are merged in the therapy suggestions.

In order to explore and compare the high number of possible therapies for some molecular profiles, Onkopus provides a unique interactive sunburst visualization of the clinical significance of a variant (Fig. 2B). In this visualization, one or more biomarkers are shown in the center, with other layers representing different characteristics of the clinical evidence, e.g. drug classification, cancer type, or match type. The outermost layer represents the clickable citation IDs of the studies that substantiate the clinical evidence, whose publication can be accessed directly via the link. The legend above each sunburst plot shows which layer represents which feature. In this case, the drug class is shown in the first layer, the drug in the second layer, the evidence level in the third layer, and the citation ID in the outermost layer.

We showcase the benefit of the Onkopus match types using the sunburst rays for finding clinical evidence on RET:p.M918T, a pathogenic variant that may occur primarily in thyroid and lung cancer tissues. For this specific variant, Onkopus yields several studies via exact match search. In addition to the search for exact matches, the search for general mutations in RET as a biomarker identifies a study investigating Selpercatinib for patients with thyroid cancer, which was classified with a sensitive response and with evidence level 1, indicating support by at least one well-powered, controlled clinical study (Fig. 2C, yellow box). The clinical evidence results may as well be grouped by the cancer type, allowing to specifically search for clinical evidence of the same tumor type (Fig. 2D) or drug classification (Fig. 2E). In case of RET:p.M918T, we can find multiple evidences for VEGFR inhibitors and RET tyrosine kinase inhibitors.

In this way, clinicians can easily get an overview of possible personalized treatments for a patient and the strength of the available evidence. The underlying idea behind generating different sunburst graphs is to present the clinical significance of variants in a molecular profile in as many different ways as possible, leaving the final treatment decision to the clinician. Onkopus automatically generates sunburst graphs associating potential therapies with single biomarkers and for all biomarkers of a molecular profile.

### Molecular and protein feature analysis

**Figure 3.**
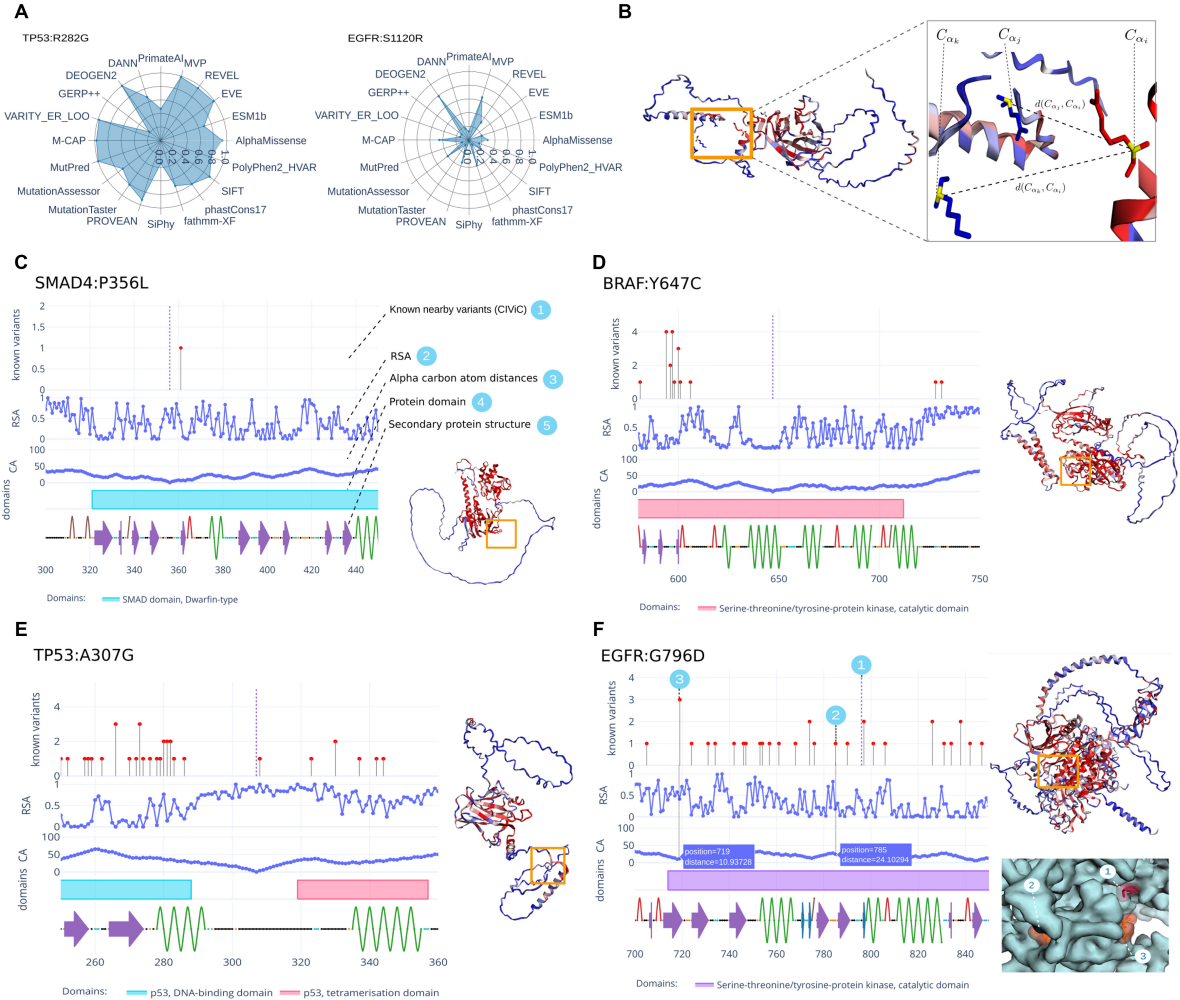
Onkopus protein analysis. (**A**) Pathogenicity predictions of different computational methods. (**B**) The alpha carbon distances are computed by calculating the distance of each position relative to the mutated site. (**C**) Onkopus protein analysis, including the visualization of nearby mutations (1), surface accessibility (2), alpha carbon distances (3), protein domains (4), and the secondary protein structure of a protein (5). Interactive 3D protein structures (mutated site in orange box) in Onkopus Web are colored according to the average AlphaMissense-predicted pathogenicity (colored blue = benign, red = pathogenic) at each position in the protein, as well as according to the binding site probability predicted by ScanNet. By analyzing the protein context of a variant, VUS can be assigned to the more benign [TP53:A307G (**E**)] or pathogenic [BRAF:Y647C (**D**) or EGFR:G796D (**F**)] spectrum. The *C*_α_ distances can help to find variants that are spatially close to the patient’s variant (**F**(1)). Due to protein folding, variants can be closer to the mutated site in 3D space (3) than variants that are closer based on the protein sequence position (2).

In order to estimate the pathogenicity of a variant, Onkopus provides an overview of the pathogenicity predictions of the most important computational methods in a preliminary step (Fig. 3A). To analyze the protein changes by a variant at the molecular level, we provide annotation with chemical features of the reference and alternate amino acid, including changes in molecular weight, charge, polarity, aromaticity, solubility, the potential of ionization, phosphorylation and ionization potential, alpha helix breakers, beta sheet propensity, and BLOSUM62 scores. In addition, we compute possible disulfide bonds and salt bridges that may occur as a result of the variant.

AlphaFold has shown remarkable performance in the Critical Assessment of Structure Prediction (CASP) competition, and the potential of using its accurate structure predictions in improving function annotation in the Critical Assessment of Function Annotation (CAFA) competition has been discussed. We have thus used AlphaFold predictions of the wild-type gene as the basis for calculating the protein structure-specific features. The distances of alpha carbon atoms within the protein are retrieved by calculating the distance of each amino acid’s alpha carbon atom relative to the mutated amino acid’s alpha carbon atom in three-dimensional space (Fig. 3B). In addition, a comprehensive array of structure-specific metrics is calculated, encompassing surface accessibility, secondary protein structure, alpha carbon distances, and hydrogen bonds. To analyze pathogenicity and binding site probability, Onkopus generates interactive 3D visualizations of the protein colored by either pathogenicity and binding sites.

In the front end, we provide an interactive, aligned visualization of the protein, including the positions of the mutated site and known variants reported in the CIViC database in the first row (Fig. 3C). The next rows visualize the amino acid’s surface accessibility, *C*_α_ distances, the protein domains, and the secondary protein structure.

To showcase how the protein analysis can help to interpret VUS, we examined selected variants reported as VUS in ClinVar. As for TP53:p.A307G (Fig. 3E), the mutated position is located in a loop structure and not affiliated with a protein domain or secondary protein structure element, while its RSA is higher than the average RSA of all amino acids within the protein. The variant is predicted as rather benign by AlphaMissense. BRAF:p.Y647C (Fig. 3D), on the other hand, is located within a protein domain, showing a low surface accessibility and a medium to high pathogenicity prediction score by AlphaMissense, indicating a rather pathogenic variant. EGFR:p.G796D (Fig. 3F) is located within the catalytic domain within an alpha helix, whereby the presence of multiple pathogenic variants in close proximity shows a hotspot here. The alpha carbon atom distances may help in identifying spatially adjacent variants: In the case of EGFR:p.G796D [Fig. 3F(1)], multiple known variants are present in the immediate vicinity, including EGFR:p.T785A (2). In the folded protein within three-dimensional space, however, amino acids may be closer to the mutated site whose sequence-based position is further away, e.g. EGFR:p.G719A (3).

### KNIME workflows

To facilitate the generation of customized variant annotation pipelines, we implemented graphical, adaptable workflows for the analytical framework KNIME. The workflows comprise a series of genome annotation nodes, each of which represent a distinct annotation, filtering, or data parsing process. The nodes may be added or combined dynamically, with each workflow commencing with a read node of the required format and concluding with one or more write nodes.

## Discussion

We presented Onkopus, a versatile tool for interpreting genetic variants that summarizes all relevant information for the biological and clinical interpretation of variants in one platform. While the majority of preliminary solutions focused on either functional or clinical annotation, Onkopus provides a more comprehensive approach that considers all aspects of variant interpretation.

Compared to other web-based variant interpretation tools, including wANNOVAR, VEP Web, REEV, and VarCards2, Onkopus provides personalized treatment options and the possibility of analyzing variants and potential therapies at patient level. Compared to commercial variant interpretation platforms such as ICI, Onkopus provides a similarly detailed overview and analysis of a patient’s molecular profile. Both offer interactive platforms for visualizing and exploring selected variants and their actionability. Since ICI is directly connected to the Illumina cloud containing sequencing data, it also includes sequencing-specific metrics, like sequencing quality and depth, coverage, or tumor mutational burden. In a clinical context, these features could also be included in Onkopus, if the framework is integrated in the clinical infrastructure. As additional features, Onkopus provides molecular and protein structural specific characteristics and is published as open source software. It aims to follow current trends in the changing field of MTBs. cBioPortal provides a comprehensive platform for cohort analysis. Customized for MTBs, it can also be used for the preparation of MTBs. Similar to Onkopus, cBioPortal includes information from external sources and relevant links to them. However, while cBioPortal is designed for cohort analysis, Onkopus focuses on finding relevant treatments for individual patients. A combination of both tools will cover both aspects of data processing. In addition, Onkopus provides more options for converting variant data to other reference genomes via LiftOver (GRCh37, GRCh38, and T2T-CHM13).

To analyze the impact of a variant on the protein structure and characteristics, several tools provide web tools to interactively analyze mutated proteins. However, these tools focus mainly on one consequence of a mutation on the protein, such as change in protein structure (Swiss-Po), or binding affinity (pSnpBind). In this way, they lack some features to view the whole context of a protein at a mutated site, such as surface accessibility, alpha carbon distances relative to the mutated site, or the molecular characteristics of amino acid exchanges. As a variant annotation framework, Onkopus provides novel annotations with molecular and structural protein features for a variant or gene, with the ability to examine the biochemical consequences of a variant at the molecular level as well as within the context of the entire protein. By combining the protein analysis with computational methods for predicting pathogenicity, MTBs can use pathogenicity scores to retrieve an initial assessment of the pathogenicity of a variant, followed by an extended analysis of the surrounding region of the mutated site.

With the visualization of possible treatments in sunburst plots and the overview of protein features, the web front end offers unique graphs to explore genetic variants and personalized therapies. In addition, Onkopus provides API endpoints for variant interpretation. Due to the modular architecture, databases can be updated very easily by only updating a single module, while the rest of the system has no downtime. All necessary modules can be installed locally, making Onkopus ideally suited for installation in clinical infrastructures with limited or no Internet access. To install the modules in a clinical infrastructure, the Onkopus command line tool offers the option of downloading all modules of the framework as packaged Docker containers and saving them in the local file system. As a stand-alone tool, Onkopus is highly flexible, and integration into other clinical frameworks could be realized via API data import.

We are confident that the dynamic architecture of Onkopus will allow for a variety of usage scenarios for variant interpretation, including the support of MTBs and genome annotation for biomedical research. This potential offers the possibility of establishing Onkopus as a widely utilized solution for variant interpretation.

## Supplementary Material

gkaf376_Supplemental_Files

## Data Availability

Onkopus is freely available under Creative Commons License (CC BY-SA 4.0). No login is required. No data are transferred to third-party partners. Uploaded data are stored anonymously. The source code of all Onkopus modules is available at https://gitlab.gwdg.de/MedBioinf/mtb/onkopus, including instructions on how to install the Onkopus modules locally. A public instance is available at https://mtb.bioinf.med.uni-goettingen.de/onkopus. Pre-built KNIME workflows are available at the KNIME Hub (https://hub.knime.com/bioinf_goe/spaces/Public/Onkopus∼C5mAjD86d6qjVWhB/). All modules have been generated from freely available data sources.
